# The association between daily 500 mg calcium supplementation and lower pregnancy-induced hypertension risk in Bangladesh

**DOI:** 10.1186/s12884-018-2046-0

**Published:** 2018-10-17

**Authors:** Fouzia Khanam, Belal Hossain, Sabuj Kanti Mistry, Dipak K. Mitra, Wameq Azfar Raza, Mahfuza Rifat, Kaosar Afsana, Mahfuzar Rahman

**Affiliations:** 1 Research and Evaluation Division, BRAC Center, Dhaka, 1212 Bangladesh; 2 Department of Public Health, North South University, Dhaka, Bangladesh; 3 Poverty and Equity, The World Bank, Dhaka, Bangladesh; 4 Health, Nutrition and Population Program, BRAC Center, Dhaka, Bangladesh

**Keywords:** Pregnancy-induced hypertension, Calcium supplementation, Maternal nutrition initiative (MNI), Global health, Maternal mortality

## Abstract

**Background:**

Evidence suggests that daily supplementation of 1500 to 2000 mg of calcium during pregnancy reduces pregnancy-induced hypertension (PIH). However, the evidence on the efficacy of low-dose calcium supplementation on PIH is limited. This paper assesses the longitudinal correlation between low-dose calcium intake (500 mg daily) and change in blood pressure during pregnancy among a homogeneous population in terms of hypertension and pre-eclampsia.

**Methods:**

The study followed a retrospective cohort study design, and was carried out among 11,387 pregnant women from 10 rural *upazilas* (sub-districts) of Bangladesh where maternal nutrition initiative (MNI), implemented by Building Resources Across Communities (BRAC), was ongoing. The modified Poisson regression model was used to estimate the association (risk ratio) between consumption of calcium tablets and PIH.

**Results:**

The present research found that women who consumed 500 mg/d calcium tablets for more than 6 months during their pregnancy had a 45% lower risk of developing hypertension compared to those who consumed less calcium (RR = 0.55, 95% CI = 0.33–0.93).

**Conclusions:**

Daily supplementation of 500 mg oral calcium during pregnancy for at least 180 tablets is associated with a considerably reduced risk of PIH, but this study is unable to confirm whether this association is causal. The causal relationship needs to be confirmed through a large scale randomized controlled trial.

**Electronic supplementary material:**

The online version of this article (10.1186/s12884-018-2046-0) contains supplementary material, which is available to authorized users.

## Background

Pregnancy-induced hypertension (PIH), defined as systolic blood pressure (sBP) > 140 mmHg or diastolic blood pressure (dBP) > 90 mmHg [1], is a major determinant of pre-eclampsia/eclampsia (PE/E). High BP is responsible for approximately 14% of global maternal deaths [2]. Pre-eclampsia affects an estimated 3.2% of all live births - a total of more than four million cases each year -nearly 1.8% of which are fatal [3–5]. First described in 1980 [6], the inverse relationship between calcium supplementation during pregnancy and the risk of pregnancy-induced high blood pressure (BP) is well documented [7–15].

Based on evidence from a meta-analysis of randomized controlled trials [16], the World Health Organization (WHO) recommends routine prenatal calcium supplementation of 1500 to 2000 mg daily beginning from 20th gestational week for all pregnant women, particularly those residing in low-calcium intake areas which are considered as high risk population [17]. It is notable that the overall calcium intake among the Bangladeshi population is low due to lack of calcium in the regular diet [18]. Although the WHO calcium regimen is endorsed by the government of Bangladesh, in reality the adoption rate has been lower due to bottlenecks such as poor compliance [19].

However, evidence of the impact of low-dose calcium supplementation (intake of 500 mg/d calcium tablet) on PIH is limited. A systematic review of the effects of low-dose calcium intake on pre-eclampsia has shown significant results [20]. The review included studies from both high-risk and low-risk populations and found a larger effect among high-risk populations accordingly. Since the data for this review came primarily from small studies, the authors called for larger trials to confirm the results.

Previous researchers have also suggested that a lower dose regimen may actually result in a higher cumulative calcium dose consumption through improved adherence [21]. High dose supplementation as recommended by WHO has not been widely adopted, likely because of practical impediments such as the size and number of units of conventional calcium tablets required to deliver the recommended daily dose (3 to 4 tablets). Furthermore, calcium tablets must be ingested separately from iron because of the negative impact of calcium on iron absorption. Therefore, building an evidence base on the effects of a low dose recommendation could have a tremendous impact in developing countries through saved resources. This study aims to assess the effects of different durations of low-dose calcium supplementation (500 mg daily) during pregnancy on the incidence of PIH.

## Methods

### Study design, sampling and participants

The study followed a retrospective cohort design. Participants were women who gave birth between November 2016 and May 2017. Basic and vital information of the pregnant mothers was extracted from the registrars of *Shashthya Kormis* (SKs) - the community health workers (CHW) working in the maternal nutrition initiative (MNI) program implemented by BRAC. All participating women received calcium tablets during the pregnancy period; therefore, there was no control group in the study.

**Fig. 1:**
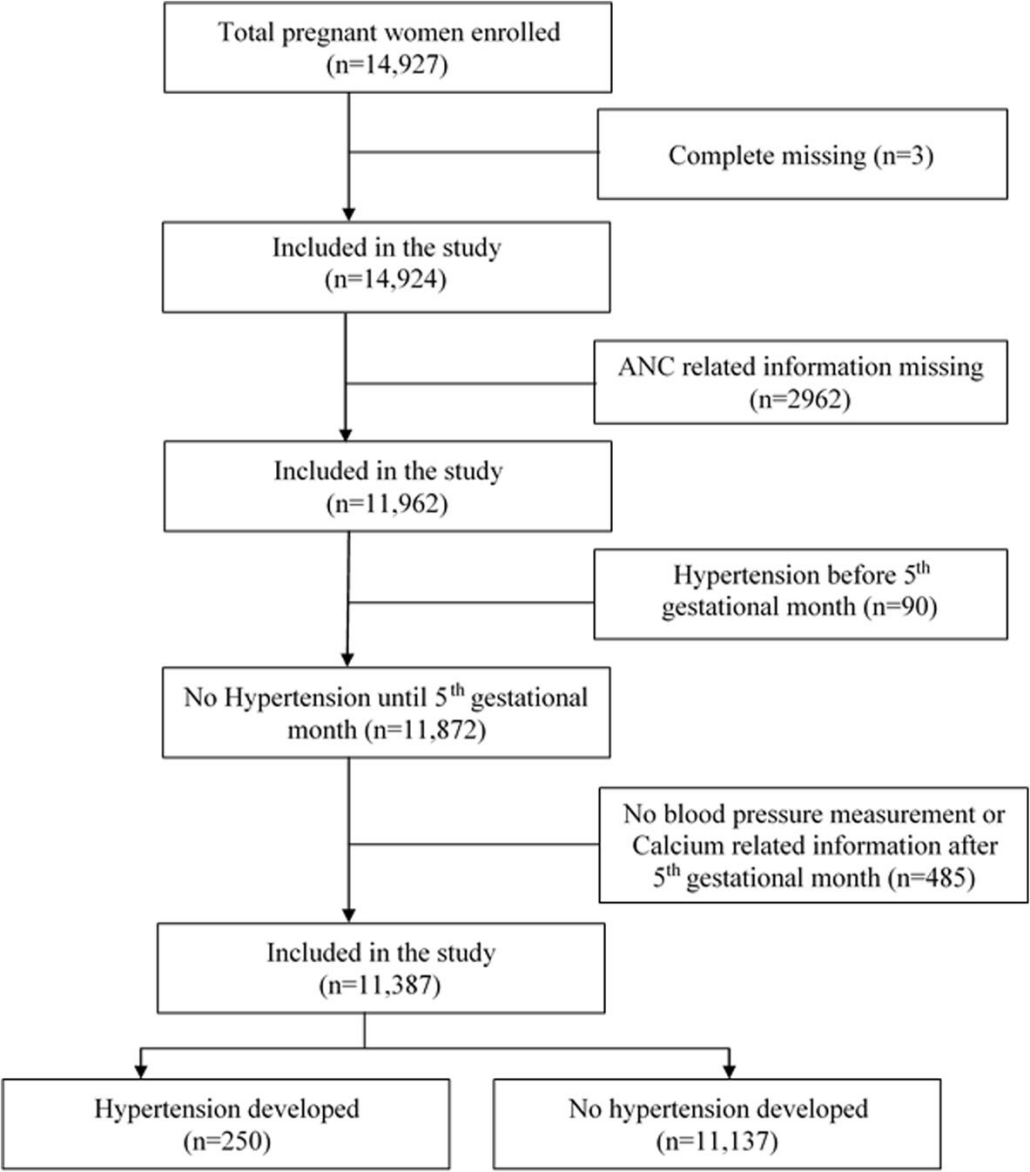
Study population and participant’s enrollment

Sample size for this study was calculated to compare incidence of PIH between women those who consumed daily 500 mg calcium for the optimal duration and women those who consumed calcium for a sub-optimal duration. Considering a 6% incidence of PIH among women with sub-optimal calcium dosing, a 4% incidence among women with optimal dosing, 5% type I error, 90% power, and a design effect of 2, a sample of 10,400 pregnant women were required for the study. We included 11,387 participants in our study. We excluded individuals with completely missing data (*n* = 3), with missing data on ANC timing (*n* = 2962), those diagnosed with hypertension before the first follow-up (*n* = 90), and those missing data on systolic blood pressure (sBP) or diastolic BP (dBP) or calcium distribution and consumption after 5 months of gestation due to lost to follow-up (*n* = 485) (Fig. 1).

### The intervention

BRAC has been implementing the maternal nutrition initiative (MNI) program in 10 upazilas (sub-district) i.e., Gafargaon, Dhobaura, Tarakanda, Trishal of Mymensingh, Badarganj and Mithapukur of Rangpur, Aditmari and Patgram of Lalmonirhat, and Rajarhat and Ulipur of Kurigram districts of Bangladesh since July 2015. The program is conducted by community health workers (CHW) which are divided into two groups - *Shashthya Kormis* (SKs) and *Shashthya Sebikas* (SSs). SKs were trained on providing different health care services such as pregnancy identification, antenatal care (ANC) and postnatal care (PNC), while SSs were selected from local communities and were trained on providing essential health care services at the community level. After identification, SKs visited every pregnant woman to provide ANC services on a monthly basis until childbirth. During consultations, SKs discussed dietary diversity, recommended high quality foods, recommended taking iron folic acid (IFA) and calcium, and collected anthropometric data including weight and blood pressure.

SKs were tasked to distributing 500 mg calcium tablets from the first ANC visit. The total number of calcium tablets received by a pregnant woman depended on gestational age at her first ANC visit. Women who began to receive ANC within the first 3 month of pregnancy received 180 tablets or more, those who came around 6 or 7 months received 90 tablets or more, and those who came at 8 or 9 months received fewer than 90 tablets. The comprehensive intervention is comprised of the following four components: (i) counseling about the importance of calcium tablet during pregnancy; (ii) free delivery of 30–35 calcium tablets (500 mg each) per month until the end of term; (iii) recording the compliance of calcium intake by counting strips of calcium tablets provided during earlier visits; and (iv) collecting anthropometric information such as weight and BP measurements.

Women were instructed to take one calcium tablet (500 mg) daily in the morning for 6 months (180 tablets) or until pregnancy termination. The daily dose was determined based on the trials included in the 2010 Cochrane review [16]. Due to concerns that calcium might interfere with IFA absorption [22], the women were instructed to take calcium tablets after their morning meal and not to take it with iron, which was to be taken with the evening meal.

### Measurement of outcome and exposure variables

Blood pressure was the primary outcome variable. BP was measured at enrollment (5th gestational month) and at each follow-up visit (6th through 9th gestational months) by trained field workers using a sphygmomanometer [23]. Measurements were taken with participants in a seated position after 5 min of rest, with the cuff around the upper left arm in accordance with recommended guidelines [24]. Two BP measurements were taken at each follow-up with a minimum 1 h interval [25], and an average of those measurements was recorded. Women were considered to have PIH if sBP ≥140 mmHg and/or dBP ≥90 mmHg in any visit after the 20th week of gestation with previously known normotensive.

Calcium consumption was the primary exposure variable. The level of calcium tablet consumption was classified into three categories: 500 mg/d for more than 6 months, 500 mg/d for 3–6 months, and 500 mg/d for less than 3 months. Weight was measured at every household visit with electronic scales (UNISCALE) accurate to 100 g. The number of living children, household structure, and monthly income were also collected at enrollment.

We validated the main outcome variable through cross-checking by medical professionals. The professionals cross-checked 105 measurements of 105 individuals in a given visit. The reliability of the BP measurements was quantified by using intra-cluster correlation coefficient [26]. We found that the intra-class correlation coefficients were very high for both systolic and diastolic BP measurements (for sBP: 0.91 and for dBP: 0.86).

### Statistical analysis

Descriptive statistics were performed to assess the distribution of the exposure variables. A Chi-squared test was conducted to compare calcium consumption groups by other exposure variables. Predicted mean blood pressure (sBP and dBP) was estimated using the mixed-effect linear regression model which accounts for the correlation among BP measurements within subjects and variations across subjects. The dependent variable of the mixed-effect model considers all successive BP measurements of individuals with complete information by considering a random intercept for each subject and BP over time in the population as fixed effect.

Finally, we investigate the unadjusted and adjusted association between calcium intake and PIH using a modified Poisson regression model assuming an uniform risk period and robust standard errors [27]. Our initial goal was to run a log-binomial regression model to assess the effectiveness of the consumption of 500 mg calcium tablets, but this model had a convergence problem with many covariates. To overcome this problem, we ran the modified Poisson regression model which is equivalent to the log-binomial regression model when estimating risk ratio (RR) [27]. The unadjusted analyses were performed for the potential confounders such as enrollment age, weight, occupation, parity, number of living child, number of antenatal care visits, household type, household assets, and administrative district. The variables with *P* < 0.25 in the unadjusted analyses were considered as confounders and included in the final model [28]. The RR and corresponding confidence interval (CI) were estimated with a 5% significance level. All analyses were performed using statistical software package STATA 13.0.

## Results

Table 1 shows the distribution of demographic and lifestyle factors among the study population. A total of 57.4% women were aged between 20 and 29 years. Most of the women (99.1%) were housewives and 76.2% women had at least one child. 14.6% women had pacca/semi-pacca households and 46.8% were from poor households. Of the total 11,387 participants 9358 had four sBP measurements and 9356 had four dBP measurements; 10,376 had three sBP and 10,377 had three dBP measurements (Table 1).

**Table 1:** Background characteristics of study participants (*n* = 11,387)

Characteristics	*n*	% or Mean ± SD
District		
Mymensingh	5649	49.61
Rangpur	2270	19.94
Lalmonirhat	1613	14.17
Kurigram	1855	16.29
Age (years)		
< 20	2733	24.00
20–24	3237	28.43
25–29	3297	28.95
≥ 30	2120	18.62
Occupation		
Housewife	11,286	99.11
Working outside	101	0.89
Parity		
1	2803	24.62
2	4452	39.10
≥ 3	4132	36.29
Number of living children		
None	2715	23.84
1	5098	44.77
≥ 2	3574	31.39
Household type		
Pacca/semi-pacca	1666	14.63
Tin and others	9721	85.37
Household asset		
Poor	5321	46.73
Non-poor	6066	53.27
Systolic blood pressure		
Baseline	11,387	99.27 ± 7.27
Follow up 1	10,151	99.94 ± 8.31
Follow up 2	10,376	100.75 ± 8.57
Follow up 3	9358	101.59 ± 9.15
Follow up 4	5224	102.41 ± 9.63
Diastolic blood pressure		
Baseline	11,387	62.50 ± 6.99
Follow up 1	10,151	63.77 ± 7.76
Follow up 2	10,377	64.52 ± 7.97
Follow up 3	9356	65.43 ± 8.48
Follow up 4	5225	66.06 ± 8.80
Weight (kg)		
Baseline	11,387	49.02 ± 8.03
Follow up 1	10,111	50.46 ± 7.99
Follow up 2	10,369	51.98 ± 8.04
Follow up 3	9363	53.58 ± 8.20
Follow up 4	5221	54.93 ± 8.28

Baseline = 5th gestational month, Follow up 1–4 = 6th – 9^th^gestational month

Table 2 shows the calcium consumption during pregnancy by demographic and socio-economic variables. A total of 19.8% women consumed < 90 tablets (i.e. 500 mg/d calcium tablets for less than 3 months), while 66.0% consumed 90–179 tablets (i.e. 500 mg/d calcium tablets for 3–6 months) and 14.2% consumed 180 or more tablets (i.e. 500 mg/d calcium tablets for more than 6 months).Women between 20 and 29 years consumed more calcium tablets than women aged < 20 or > 30 years (*P* < 0.001). Low body-weight women (< 45 kg at enrollment) consumed a higher number of calcium tablets than higher body-weight women (*P* = 0.105). Women living in pacca/semi-pacca households consumed more tablets (*P* = 0.047), while consumption was similar between poor and non-poor households (*P* = 0.794). Moreover, calcium consumption largely depended on the number of ANC visits. Women who had four or more ANC check-ups consumed more calcium tablets than those who did not (*P* < 0.001). However, among all 11,387 study women, all had at least one ANC visit while 86% had four or more (Additional file 1: Table S1).

**Table 2:** Background characteristics of women by calcium consumption level during pregnancy (*n* = 11,387)

Characteristics	*n*	500 mg/d calcium intake (%)			*P*
		< 3 months	3–6 months	≥6 months	
Age (years)					< 0.001
< 20	2733	21.08	66.26	12.66	
20–24	3237	19.56	64.94	15.51	
25–29	3297	17.26	67.52	15.23	
≥ 30	2120	22.36	65.09	12.55	
Occupation					0.006
Housewife	11,286	19.68	66.16	14.16	
Working outside	101	30.69	51.49	17.82	
Baseline weight (kg)					0.105
< 45	3585	20.00	64.44	15.56	
45–50	3571	19.46	67.04	13.50	
51–60	3171	19.55	66.98	13.47	
> 60	1060	20.75	65.19	14.06	
Parity					0.006
1	2803	20.91	65.93	13.16	
2	4452	18.60	65.90	15.50	
≥ 3	4132	20.28	66.24	13.48	
Number of living children					< 0.001
None	2715	21.22	66.08	12.71	
1	5098	18.24	66.40	15.36	
≥ 2	3574	20.87	65.47	13.65	
Number of antenatal care visits					< 0.001
< 4	1295	86.64	13.36	0.00	
4+	10,092	11.20	72.79	16.01	
Household type					0.047
Pacca/semi-pacca	1666	21.49	65.85	12.67	
Tin and others	9721	19.48	66.06	14.45	
Household asset					0.794
Poor	5321	19.51	66.23	14.26	
Non-poor	6066	20.01	65.86	14.13	
Total	11,387	19.78	66.03	14.19	

Table 3 shows the predicted mean sBPs and dBPs at baseline and four follow up visits by calcium intake categories. It also presents the absolute change in mean blood pressures from baseline to the last follow up visit. For both sBP and dBP, we observed a slower increase over time among those who consumed 500 mg/d calcium tablets for more than 6 months during the pregnancy period compared to those who consumed 500 mg/calcium tablets for fewer than 6 months.

**Table 3:** Relation between number of calcium tablets intake and monthly changes in systolic blood pressure (sBP) and diastolic blood pressure (dBP) over 4 months of follow-up (5th to 9th gestational months) of the same individuals

Characteristics	Baseline	F1	F 2	F3	F4	BP increase
sBP (mmHg)						
Calcium intake						
500 mg/d for < 3 months	96.7	98.2	99.2	100.4	102.4	5.7
500 mg/d for 3–6 months	98.9	99.6	100.4	101.1	102.2	3.4
500 mg/d for ≥6 months	99.6	100.5	101.1	101.5	102.7	3.1
dBP (mmHg)						
Calcium intake						
500 mg/d for < 3 months	62.0	62.6	63.0	64.7	67.0	5.0
500 mg/d for 3–6 months	62.5	63.0	63.9	64.7	65.9	3.4
500 mg/d for ≥6 months	63.2	63.9	64.3	65.1	65.8	2.7

Baseline = 5th gestational month, F1 to F4 = Follow up 1 to 4 (6th to 9th gestational month)

We further compared the incidence of PIH after the 5th gestational month between calcium intake groups using log-binomial or modified Poisson regression model. Both unadjusted and adjusted models were implemented and results are presented in Table 4. The final model was adjusted by age and weight at enrollment, number of antenatal care visits, household asset, and administrative district. The overall incidence of PIH was 2.2% (250 out of 11,387). We found that women who consumed 500 mg/d calcium tablets for more than 6 months during the antenatal period had a significantly lower risk (46%) of developing hypertension than those who consumed 500 mg/d calcium tablets for fewer than 3 months (RR: 0.56, 95% CI: 0.33–0.93).

**Table 4:** Relation between amount of calcium intake and pregnancy-induced hypertension

Characteristics	Unadjusted			Adjusted		
	RR	*P*	95% CI	RR	*P*	95% CI
Calcium intake						
500 mg/d for < 3 months	1.0			1.0		
500 mg/d for 3–6 months	0.78	0.089	0.59–1.04	0.83	0.273	0.59–1.16
500 mg/d for ≥6 months	0.51	0.005	0.32–0.82	0.56	0.026	0.33–0.93
Age (years)	1.04	0.001	1.02–1.06	1.02	0.065	1.00–1.05
Weight (kg)	1.07	< 0.001	1.06–1.08	1.06	< 0.001	1.05–1.07
Occupation						
Housewife	1.0			Not retained in the final model		
Working outside	0.74	0.594	0.24–2.26			
Parity						
1	0.96	0.802	0.70–1.32	Not retained in the final model		
2	1.0					
≥ 3	1.08	0.601	0.81–1.43			
Number of living children						
None	1.0			Not retained in the final model		
1	1.05	0.781	0.76–1.44			
≥ 2	1.14	0.444	0.82–1.59			
Number of antenatal care visits						
< 4	1.0			1.0		
4+	0.72	0.054	0.51–1.01	0.91	0.646	0.60–1.37
Household type						
Pacca/semi-pacca	1.0			Not retained in the final model		
Tin and others	0.87	0.423	0.63–1.22			
Household asset						
Poor	1.0			1.0		
Non-poor	1.36	0.016	1.06–1.75	1.14	0.307	0.88–1.48
District						
Mymensingh	1.0			1.0		
Rangpur	2.05	< 0.001	1.52–2.77	1.84	0.000	1.36–2.50
Lalmonirhat	1.69	0.004	1.19–2.42	1.56	0.013	1.10–2.23
Kurigram	1.34	0.121	0.93–1.93	1.18	0.378	0.82–1.70

*RR* Risk ratio, *CI* Confidence interval

## Discussion

To our knowledge, this large-scale study is the first to examine the effectiveness of low dose supplemental calcium (500 mg) on the risk of PIH. We found that women had a 45% lower risk of developing hypertension when they took 500 mg/d calcium tablets for more than 6 months during pregnancy relative to those who consumed 500 mg/d calcium tablets for fewer than 3 months. The association of calcium consumption during pregnancy with decreased PIH is well documented in earlier studies [20, 29], yet the evidence of the use of low-dose calcium in a maternal health and nutrition intervention is scarce in low-income settings. We begin to fill this gap in the literature.

The World Health Organization (WHO) has recommended antenatal calcium supplementation of 1500–2000 mg daily for pregnant women with low dietary calcium intake who are thus at a higher risk for pre-eclampsia [16, 30]. However, a large proportion of Bangladeshi pregnant women are at high risk for multiple micronutrient deficiencies including antioxidants [18, 31]. While much research has focused on malnutrition among children, recent reports indicate a high prevalence of micronutrient deficiency among women as well [32]. Under these conditions, BRAC initiated the MNI program which includes the 500 mg daily calcium supplement.

Our study found that women living in the Rangpur and Lalmonirhat districts are at higher risk of PIH compared to Mymensingh district when adjusting for other factors including calcium intake. This result is consistent with the fact that Rangpur division has a higher prevalence calcium deficiency compared to the Dhaka division [18]. We also found that older age and higher weight significantly increased the risk of PIH which is consistent with findings from other studies [33].

Nevertheless, cross-sectional assessments of the association between calcium exposure and BP are limited by *a*) possible selection bias in capturing only individuals who have lived long enough to participate in the study, and *b*) weak detection of the latent effects of calcium exposure on BP. In contrast, longitudinal analyses mitigate some of these problems and may be a robust method for examining the effect of calcium consumption on blood pressure change over time.

In the present study, we found that 500 mg/d calcium tablets consumption for 180 days or more during pregnancy was associated with lower increase in mean BP. Also, the risk of hypertension was 45% lower among those women. While the observed reduction is significant, it does not point directly to clinical outcomes at the individual level. A majority of the women were lost from the sample before receiving ANC visits and calcium tablets, thus resulting in a type of survival bias. Subsequent loss at follow up resulted in missing outcome data. However, as our study population is very homogenous in nature, we expect very little bias from this loss. One major strength of our study is that any biases were minimized by combining individual calcium consumption and outcome data from registry. Moreover, loss at follow-up or missing data is a problem regardless of any possible assuring comparisons. We simply do not know how dropped observations could have affected risk ratios were they not missed. Moreover, most of the previous research which has been undertaken to assess the effectiveness of low-dose calcium was carried out among populations at high risk for calcium deficiency. In contrast, the present research was more robust in the sense that the data were collected from a general group of population, i.e. there was no particular low or high risk population.

The study is not without limitations. First, the conclusions are drawn in the absence of full-fledged randomized control trial. Due to lack of a true comparison group - especially the absence of a high dose supplementation group - we are unable to attribute the effectiveness with greater strength. We also did not collect the information through a questionnaire; rather information was extracted from the registrars of the SKs who were already working in the MNI areas. The registrars did not contain much relevant information which may influence both the calcium intake and blood pressure level among the women. Therefore, the study result should be used with caution as the information of few confounders such as body mass index, diabetes, family history of PIH and previous incident of PIH were missing [34, 35]. We are also unable to account for measurement errors as only one method of measurement was employed throughout the study duration.

Also, because the MNI initiative included the improvement of the dietary practices of pregnant and lactating mothers, the observed reduction in PIH could be due to an increase in calcium intake resulting from a generally improved diet. We did not assess the role of specific nutrients or nutritional intake in the present study. Future studies are needed to investigate whether the association of calcium intake and the rate of BP change differ by nutritional status.

## Conclusion

The study demonstrated a positive association between low dose calcium supplementation and reduction in risk of BP among a large cohort of pregnant women who were homogeneous with respect to disease risk. The findings have several policy implications. These findings are consistent with other studies about reducing the risk of PIH which could have implications for current guidelines and their global implementation. Currently, the high cost of implementing the WHO recommended daily dose of calcium is regarded as prohibitive in low income settings. Moreover, this dose has not been widely adopted due to implementation bottlenecks, such as the difficulty of multiple (3–4 tablets) administrations per day. Therefore, the dilemma facing health policy-makers in these settings is often whether supplementation with a lower-dose would be better than no supplementation at all. The findings of this research are a step towards addressing the issue.

Currently, the introduction of calcium supplementation in maternal health program is strongly recommended by WHO especially among populations with low dietary intake of calcium. So farm knowledge is inadequate concerning adherence, motivation, costing and logistics to implement the scaling-up of public health program. Therefore, larger well-designed RCTs are still required to determine the efficacy of low dose calcium of 500 mg/d and to determine the optimal duration of supplementation. Future research should focus on the causal impact of these interventions as well as implementation research to find out optimal program strategies including cost-effectiveness.

## Additional files

Additional file 1: Coverage of antenatal care among the surveyed women. (DOCX 13 kb)

Additional file 2: Calcium_data.CSV. The data contain the information of women who gave birth between November 2016 and May 2017. The information were on age (years), occupation, weight at baseline (kg), parity, number of living children, number of antenatal care visits, household type, household asset, administrative district, intake of calcium tablets during pregnancy period, and pregnancy-induced hypertension. (CSV 949 kb)

## Abbreviations

ANC: antenatal care
BP: blood pressure
BRAC: Building Resources Across Communities
CHW: community health worker
dBP: diastolic blood pressure
IFA: iron folic acid
MNI: maternal nutrition initiative
PE/E: pre-eclampsia/eclampsia
PIH: pregnancy-induced hypertension
PNC: post natal care
sBP: systolic blood pressure
SK: shashthya kormi
SS: shashthya sebika
WHO: world health organization
