# Supplementary material for: The association between daily 500 mg calcium supplementation and lower pregnancy-induced hypertension risk in Bangladesh
Source: BMC Pregnancy Childbirth. 2018 Oct 17;18:406. doi: 10.1186/s12884-018-2046-0 (PMC6192122; doi:10.1186/s12884-018-2046-0)
Supplement: Supplementary file 1 — Coverage of antenatal care among the surveyed women. (DOCX 13 kb) [file 12884_2018_2046_MOESM1_ESM.docx]

**Table S1: Coverage of antenatal care (ANC) among all surveyed women (N=11,387)**

| Characteristics | n | % |
| --- | --- | --- |
| Attendant at least one ANC visit | 11387 | 100.0 |
| Attendant at least four ANC visits | 10092 | 88.63 |
| Attendant at least one ANC within 4^th^ gestational month | 8817 | 77.43 |
